# Diagnostic testing for chronic spontaneous urticaria with or without angioedema: The do's, don't and maybe's^☆^

**DOI:** 10.1016/j.waojou.2025.101068

**Published:** 2025-06-09

**Authors:** Jonathan A. Bernstein, Ignacio Ansotegui, Riccardo Asero, Aleena Banerji, Stephen Betschel, Timothy Craig, José Luis García-Abujeta, René Maximiliano Gómez, Anete S. Grumach, Michihiro Hide, David M. Lang, Michael Levin, Hilary J. Longhurst, Marcus Maurer, Mario Morais-Almeida, Dinh Nguyen Van, Helena Pité, Marc A. Riedl, María Isabel Rojo Gutiérrez, Sarbjit S. Saini, Gordon Sussman, Elias Toubi, Andrea Zanichelli, Bruce Zuraw, Torsten Zuberbier

**Affiliations:** a University of Cincinnati College of Medicine, Department of Internal Medicine, Division of Rheumatology, Allergy and Immunology, 231 Albert Sabin Way, ML#563, Cincinnati, OH, 45267-0563 United States; b Hospital Quironsalud Bizkaia, Bilbao, Spain; c Clinica san Carlo, Paderno Dugnano, Milan, Italy; d Massachusetts General Hospital, Boston, MA, United States; e St. Michaels Hospital, University Health Network Toronto, and University of Toronto, Toronto, Ontario, Canada; f Penn State University, Hershey, PA, United States; g University Hospital San Juan de Alicante, San Juan de Alicante, Spain; h Catholic University of Salta, Salta, Argentina; i University Center Health ABC, Santo Andre, São Paulo, Brazil; j Hiroshima Citizens Hospital and Hiroshima University, Hiroshima, Japan; k Cleveland Clinic Respiratory Institute, Cleveland, OH, United States; l Red Cross Children's Hospital, University of Cape Town, Cape Town, South Africa; m University of Auckland, Department of Medicine, Auckland City Hospital, Grafton, Auckland, New Zealand; n Allergie-Centrum-Charité, Charité – Universitätsmedizin, Berlin, Germany; o CUF Descobertas Hospital, Lisbon, Portugal; p Vinmec Institute of Immunology, College of Health Sciences, Vin University, Center of Allergy and Clinical Immunology, Vinmec Times City Vinmec Healthcare System, Hanoi, Vietnam; q CUF Tejo Hospital, NOVA Medical School, Universidate NOVA de Lisboa, Lisbon, Portugal; r University of California San Diego School of Medicine, La Jolla, CA, United States; s Hospital Juárez de México, Mexico City, Mexico; t Johns Hopkins University School of Medicine, Baltimore, MD, United States; u University of Toronto, Division of Clinical Immunology and Allergy, Toronto, Ontario, Canada; v Bnai-Zion Medical Center, Technion, Haifa, Israel; w University of Milan, Angioedema Center, IRCCS Policlinico San Donato, Milan, Italy; x US HAEA Angioedema Center at UCSD, Division of Allergy & Immunology, Department of Medicine University of California San Diego, San Diego, CA, United States; y Charité Universitätsmedizin Berlin, Berlin, Germany

**Keywords:** Chronic spontaneous urticaria, Angioedema, Biomarkers, Phenotypes, Endotypes, Treatment responders

## Abstract

Chronic spontaneous urticaria (CSU), with or without angioedema, is heterogeneous and comprised of different endotypes and phenotypes. Because acute urticaria will mostly resolve spontaneously, routine testing and laboratory evaluation is not required unless supported by the clinical history or physical examination. With the advent of omalizumab, there has been a surge of interest in identifying biomarkers that could predict response to this treatment. In the process of investigating biomarkers as prognosticators, several CSU phenotypes and endotypes have emerged, which have made it evident that novel therapies targeting non-IgE mechanistic pathways are needed to control symptoms in patients unresponsive to the currently recommended therapies by the most recent international guidelines. The current data support peripheral eosinophils, autoantibodies against IgE or FcεRI α subunit measured by basophil histamine release assays, total IgE levels and IgG autoantibodies against thyroid peroxidase (TPO) as specific markers to differentiate type 1 autoimmune (autoallergic) CSU from type 2b autoimmune CSU before starting treatment especially with omalizumab. These markers have been included as exploratory endpoints in many clinical trials investigating novel therapies or for repurposing existing biologics to determine responders and non-responders, but these data are not completely clear at this time. Therefore, further randomized controlled studies and real-world studies are needed to demonstrate more conclusively the utility of ordering these tests in CSU patients when they initially present or when it is determined they are not responsive to high dose second generation H1-antihistamines (SGAH) before they can be included in evidence-based CSU guidelines. This review examines the value of obtaining diagnostic tests in the initial evaluation of CSU patients to predict treatment response.

## Introduction

Chronic spontaneous urticaria (CSU), with or without angioedema, is heterogeneous and comprised of different endotypes and phenotypes. While these may appear similar, there are clues that merit consideration for their identification. To be confident, we are proposing appropriate diagnostic evaluation and management based on the best evidence.^1^ There is no reference standard for diagnosis of chronic urticaria. The consensus definition for the clinical diagnosis of CSU is the presence of itchy wheals (hives), recurrent angioedema, or both.^2^ In CSU, wheals are polymorphic, have serpiginous borders, are evanescent (typically lasting for less than 24 h), and recur continuously or intermittently for more than 6 weeks in a range of intensity.^2^

Current guidelines for urticaria management recommend a diagnostic work-up that focuses on a comprehensive history and physical examination^1^ — including identification of triggers and underlying causes, differential diagnosis with urticaria-like conditions, disease activity modifiers, comorbidities, consequences, predictors of the course of disease, physical examination to detect abnormalities such as thyromegaly or organomegaly and the morphology of urticarial lesions, response to treatment, and the assessment of disease activity, control, and impact on quality of life.^3^^,^^4^

Because acute urticaria will mostly resolve spontaneously, routine testing and laboratory evaluation is not required unless supported by the clinical history or physical examination.^5^ The initial steps include a complete history assessing frequency, circumstances of onset, duration as well as the presence of local or systemic symptoms that can determine whether there is any specific trigger.^6^^,^^7^ A history should rule out anaphylaxis which would involve other organ systems beyond urticaria and angioedema. If the history suggests a specific causative agent such as a drug or food, then appropriate testing which may include skin or serologic testing to the suspected agent followed by provocation to exclude or confirm this should be conducted.

However, empiric elimination diets (not guided by history and testing) and extensive laboratory evaluation are not recommended in the diagnosis of urticaria.^5^

If acute urticaria progresses to chronic disease and inducing factors cannot be identified, a complete differential blood count, erythrocyte sedimentation rate and/or C-reactive protein (CRP), and thyroid stimulating hormone (TSH) has been recommended.^5^ Extensive routine testing does not lead to improved outcomes of care and is not cost-effective.^8^^,^^9^ However, more recently, in the clinical setting, a number of serum biomarkers have been suggested for identifying more difficult to treat chronic autoimmune urticaria (Table 1) and several have been correlated with good or poor response to omalizumab including total IgE and IgG-anti-thyroid peroxidase autoantibodies (IgG anti-TPO) (Table 2).^1^^,^^3^^,^^10^^,^^11^

**Table 1:** Summary of biomarkers and clinical features of chronic autoimmune spontaneous urticaria (CAU).

Biomarkers/clinical features		Type I aiCSU (Autoallergic CSU)	Type IIb aiCSU (Autoimmune CSU)		Notes
			Anti-FcεRI	Anti-IgE	
Biomarkers available in daily practice	Total IgE	Low in patients without and with increased IgE-anti-TPO levels	Low; <30–40 IU/ml		
	Total IgA		Low; <1.84 or 0.7 g/L		
	Blood basophil counts	Might be increased in patients with elevated IgE-anti-IL-24	Decreased; <0.01 × 10^9^/L		
	Blood eosinophil counts	Unknown	Decreased; <0.05 × 10^9^/L		
	anti-TPO IgG levels	Usually negative but might be high in patients with increased anti-TPO IgE levels	High; in 39–62%		
Biomarkers measurable in specified laboratories	IgE to autoantigens	Against TPO, IL-24, ds-DNA, EPO, etc.	–	–	
	IgG to autoantigens (preferably shown by biological assay; basophil activation test and/or basophil histamine release assay)	–	Against FcεRI	Against IgE	IgG detected by biochemical assay, such as ELISA, may not be functional and may be present in healthy individuals. Other isotype autoantibodies (i.e. IgA, IgM, IgE) may also be detected in certain patients.
Clinical test	Autologous serum skin test		Positive		High sensitivity, low specificity
Response to treatments	Antihistamines	Usually good but might be poor in anti-TPO IgE-positive cases	Poor		Several studies showed no difference between patients with and those without IgG against FcεRI^7^.
	Omalizumab	Expected to be fast and good in patients with anti-TPO IgE	Slow and poor		
	Cyclosporine	Unknown	Good		
Other clinical characteristics	Disease activity/control		Poor		
	Sex		High female rates; females comprise 87–93% of cases		
	Rates of allergic diseases	Might be higher in patients with anti-TPO IgE	Unknown		

TPO; thyroid peroxidase, EPO; eosinophil peroxidase, ds; double strand, ELISA; enzyme-linked immunosorbent assay.

Modified from Kolkhir P, et al. J Allergy Clin Immunol 2022, 149:1819–1831.

**Table 2:** Biomarkers associated with natural course, severity and response to treatment in patients with chronic spontaneous urticaria.

	Shorter duration	High disease activity	Longer duration	Refractory to antihistamines	Responsive to omalizumab	Responsive to cyclosporin
Elevated IgE	X				X	
Elevated CRP	X	X		X		
Positive ASST		X		X		X
Positive IgG anti-TPO			X			
IL17, 33		X				
IL-31		X (Itch)				
Low IgE						X

IL = interleukins, ASST = autologous serum skin test, TPO = thyroid peroxidase, CRP C-reactive protein.

**Fig. 1:**
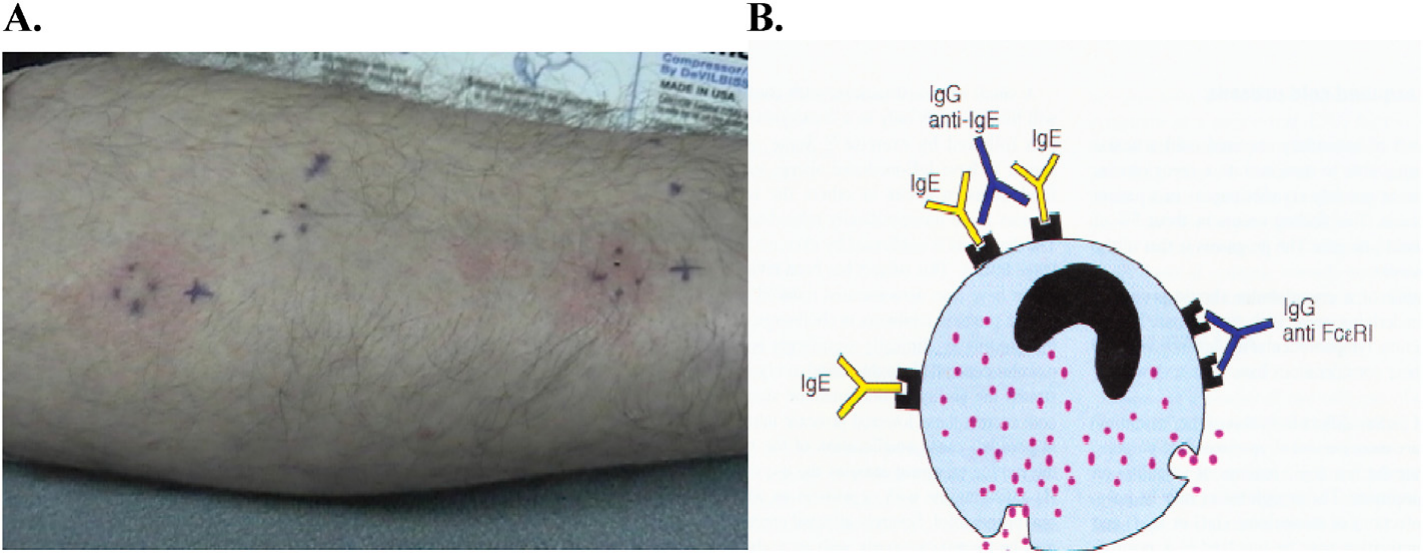
**A. In vivo Autologous Serum Skin Test (ASST) Demonstrating FcεRI Antibody. B. IgG antibody to α subunit of FcεRI (35–40%), IgG antibody to IgE (5–10%) of all CSU cases∗**. ∗ The ASST is a surrogate marker for autoantibody targeting FcER1alpha subunit or IgE attached to the receptor and therefore is a non-specific test. The CU index is an *in vitro* basophil histamine release assay in which a patient's serum with presumed IgG-anti-IgE or IgG anti-FcER1 is mixed with donor basophils and the released histamine levels are measured through a quantitative enzyme immunoassay

The autologous skin serum test (ASST) (Fig. 1), immunoassays for IgG, anti-IgE and anti-FcεRI, and basophil activation assays, by which the presence of serum histamine-releasing factors (including but not limited to IgE and FcεRIα-specific autoantibodies) can be detected by measuring histamine release from basophils (basophil histamine release assay) or by measuring upregulation of the basophil marker CD203c using flow cytometry in response to sera of patients with CSU (basophil activation test, BAT),^12^ are currently the only available tests for assessing mast cell-activating autoantibodies, but are not yet validated or widely commercially available outside of the United States.^6^^,^^7^ With the advent of omalizumab, there has been a surge of interest in identifying biomarkers that could predict response to this treatment. This has resulted in several studies suggesting additional testing may be justified prior to starting therapy with high dose second generation antihistamines (SGAH), omalizumab or cyclosporine that could be used to provide CSU patients with realistic expectations regarding treatment responses to these different therapies.^13^^,^^14^ In the process of investigating biomarkers as prognosticators, several CSU phenotypes and endotypes have emerged, which have made it evident that novel therapies targeting non-IgE mechanistic pathways are needed to control symptoms in patients unresponsive to the currently recommended therapies by the most recent international guidelines. This review examines the value of obtaining diagnostic tests in the initial evaluation of CSU patients to predict treatment response.

### Case report

A 25-year-old male presents with chronic urticaria and angioedema especially involving the lip and eyes for 3 months. He has not noted triggers. His wheals are present with most episodes of angioedema but the angioedema can occur without “hives”. He has been taking up to 40 mg of cetirizine a day and despite this has remained poorly controlled. He presents to your office for management of recurrent CSU and angioedema despite treatment with high doses of SGAHs. His exam is normal except for inducible urticaria testing that is positive for symptomatic dermographism. Based on the WAO International Guidelines your next step would be to consider starting omalizumab.^15^ Your initial workup included a comprehensive history and physical exam and basic laboratory testing which included a CBC with differential revealing a low eosinophil count and high mean platelet volume, a high C-reactive protein and normal TSH. Based on these findings, additional testing including a total IgE, thyroid peroxidase level, d-dimer level and chronic urticaria index may be appropriate.

## Chronic spontaneous urticaria: Phenotypes and endotypes

**Fig. 2:**
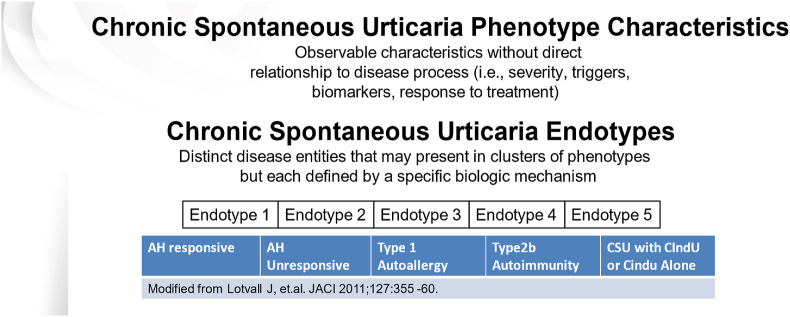
The Chronic Spontaneous Urticaria Phenotypes/Endtypes.^17^

**Fig. 3:**
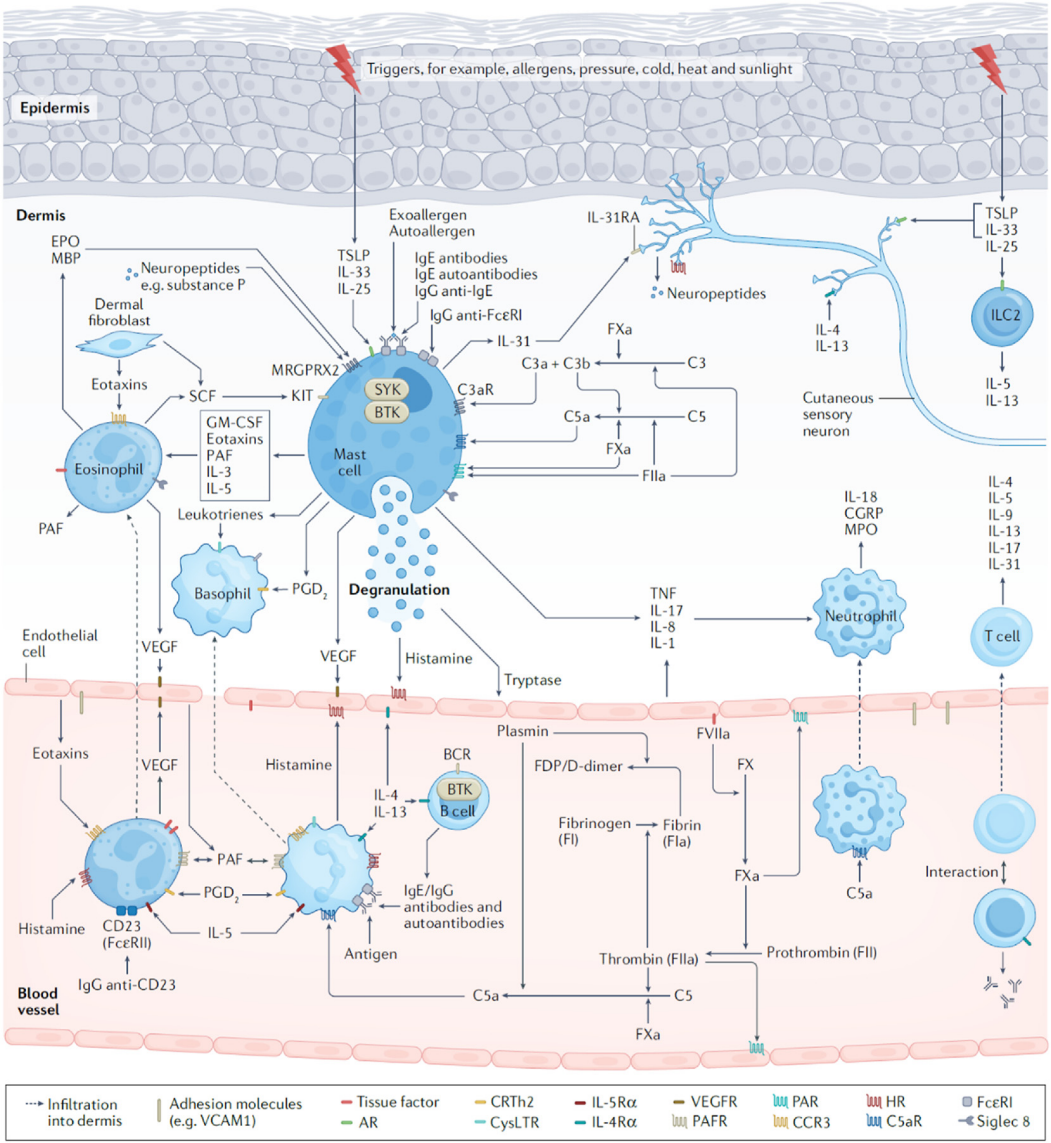
Pathophysiology Of Chronic Spontaneous Urticaria And Potential Therapeutic Targets.^16^

Chronic spontaneous urticaria can be regarded as a syndrome with multiple phenotypes and endotypes characterized by different mechanisms of action, clinical features and differential responses to treatment. Subsequent to omalizumab's approval for CSU, we have learned about responder and non-responder phenotypes and corresponding mechanistic endotypes.^16^ Fig. 2 illustrates some of the subtypes of CSU with different biologic mechanisms that have varying characteristics, triggers, and biomarkers.^17^ Mast cells are believed to be the critical effector cell type involved in CSU and chronic inducible urticarias (CIndU).^18^, ^19^, ^20^ Mast cells express several receptors, in addition to FcεRI, that have been targeted by novel therapies.^21^, ^22^, ^23^ Activating receptors include mas-related gene peptide receptor (MRGPRX2), complement receptors (C5aR), protease activated receptors (PAR1, PAR2) and chemoattractant receptor-homologous molecule expressed on T helper 2 cells (CRTh2).^24^ Inhibitory receptors include sialic acid-binding immunoglobulin-like lectin 8 (Siglec-8), Siglec-6, CD200R, CD300a, and FcγRIIb, which silence mast cells and block mast cell activation by interacting with specific ligands (Fig. 3).^24^ Unfortunately, repurposing of biologics approved for asthma, and other allergic and non-allergic conditions have been disappointing when studied for CSU or CIndU with the exception of dupilumab that has completed clinically effective phase 3 trials and is awaiting approval for CSU by the US Food and Drug Administration (FDA).^25^, ^26^, ^27^

Various CSU subtypes of both immunologic and non-immunologic pathways are characterized by differing cellular infiltrates, involvement of the coagulation and complement pathways, autoantibodies against self-antigens or FcεRI α subunit, neurogenic pathways and inducible triggers.^16^ For example, CSU characterized by eosinopenia and basopenia is observed in 0–15% of patients with urticaria and is associated with increased CSU activity, presence of autoantibodies and poor response to SGAH and omalizumab.^28^, ^29^, ^30^, ^31^, ^32^, ^33^, ^34^ Patients with biopsies comprising mixed cell infiltrates of eosinophils and neutrophils are associated with autoimmune CSU and are also less responsive to H1-antihistamines and omalizumab.^35^ In some patients with neutrophilic infiltrates on biopsy, autoimmune disorders (neutrophilic dermatoses) may be present that are more responsive to medications like dapsone, hydroxychloroquine or colchicine.^36^ Furthermore, angioedema and CIndU have been identified as risk factors for more severe and prolonged course of CSU, respectively.^16^ Activation of coagulation and fibrinolysis is associated with increased mean platelet volume, D-dimer, fibrin and fibrinogen degradation products, prothrombin fragment 1 + 2, FVIIa, amyloid serum S and other molecules.^16^ Several studies have linked increased D-dimer to severe CSU which decreases during remission.^16^ In fact, one-half of patients with severe CSU refractory to high dose SGAH, have elevated D-dimer plasma levels,^37^, ^38^, ^39^ which parallels disease activity and treatment response. Chronic spontaneous urticaria patients with increased C-reactive protein (CRP) levels also may exhibit increased D-dimer levels, IL-6, C3 and C4 levels, more severe CSU activity and autologous serum skin test positivity consistent with type 2b autoimmune CSU.^16^ For example, when autoantibodies bind to IgE or to the α-subunit of FcϵRI on mast cells and basophils, these cells are activated, and release preformed and newly formed bioactive mediators.^16^^,^^31^^,^^32^ In type I autoimmune CSU, also called autoallergic CSU, patients exhibit IgE to self-antigens such as thyroid peroxidase (TPO), IL-24, or transglutaminase 2.^16^^,^^28^^,^^29^^,^^40^ There is now evidence suggesting that type 1 autoimmunity and type 2b autoimmunity overlap as there may be co-expression of IgG and IgE autoantibodies in the same patient.^41^ Thus, it is apparent for CSU the adage “One Size Fits All” does not apply. Whether biomarkers will serve as useful clinical tools for directing clinical treatment decisions requires further investigation to confirm.

## To test or not to test patients with chronic urticaria, that is the question

The presence of angioedema, longer duration of CSU and concomitant CIndU are all associated with more severe urticaria. The limited testing that has been suggested by the international guidelines and the AAAAI Practice Parameter Update 2014 (A-PP) includes a CBC with diff, ESR or CRP, liver enzymes and TSH.^3^^,^^5^ However since the approval of omalizumab for the treatment of CSU, high IgE and IgG-anti-TPO, a low total IgE and positive basophil releasing histamine release assay or BAT that correlate with autoantibodies directed against FcεRI have been found to correlate with delayed or lack of salutary response to omalizumab suggesting these tests may be useful for making treatment decisions if patients are not responsive to Step 1 therapy.^13^^,^^33^^,^^42^

The current approach for limited diagnostic testing recommended by A-PP liver enzymes, largely based on a study by Tarbox et al which demonstrated that extensive diagnostic testing was not associated with improved outcomes.^5^^,^^9^ However, evidence concerning responders and non-responders to omalizumab and other therapies including SGAHs and cyclosporine has advanced in recent years, and these data have fostered an understanding that, rather than directing a search for a cause for urticaria, diagnostic testing has utility for predicting response to treatment and long-term prognosis.^3^

Another notable recommendation in the latest guideline is the use of patient reported outcome measures to assess severity and control.^3^ The most recent chronic urticaria guideline disseminated by EAACI, GA2LEN, Euroguiderm, and APAAAC, directs therapy using the Urticaria Control Test (UCT),^3^^,^^43^ a four-question validated instrument that queries about physical impact of hives, quality of life and therapeutic control. A score of 16 (complete control) suggests the possibility of stepping down on therapy whereas a score of 12–15 (well controlled disease) suggests therapy should be continued but optimized and a score below 12 (poorly controlled disease) suggests step-up therapy is indicated.^3^^,^^43^ If patients are unresponsive to H1-antihistamines, phenotyping and biomarkers can be used to anticipate severity, duration, and response to therapy. For example, higher levels of total IgE suggest a shorter time to relapse if therapy is discontinued and a better response to omalizumab.^44^ An elevated CRP, in addition to suggesting an infection, correlates with urticaria activity and quality-of-life impairment, as well as inflammatory and coagulation markers,^27^ and CRP is significantly higher in patients unresponsive to H1-antihistamines.^45^ Anti-TPO IgG and/or IgE antibodies suggest autoimmunity, which represent different phenotype/endotypes but can overlap, and are associated with more prolonged disease.^11^^,^^46^ Positive basophil activation tests (BAT) suggest increased disease severity and poorer or slower response to omalizumab.^47^^,^^48^ Patients expected to respond to omalizumab often have high levels of IgE, while low IgE and positive ASST suggests good response to cyclosporin.^3^^,^^22^^,^^31^^,^^32^^,^^49^, ^50^, ^51^, ^52^, ^53^, ^54^, ^55^, ^56^, ^57^, ^58^, ^59^ (Table 2). Finally, elevated levels of IL-17, IL-31 and IL-33 define patients who may have more severe pruritus and increased disease activity.^54^

Although the most recent guideline discusses biomarkers for making treatment decisions, they fall short of adding these diagnostic tests into the treatment algorithm. Prior to supporting definitive recommendations for use of biomarkers to make therapeutic decisions, validation of these different tests is required.^3^

## Should skin biopsy be obtained for patients with poorly controlled CSU unresponsive to high dose second generation antihistamines before initiating omalizumab?

Generally, skin biopsies have been traditionally performed in CSU for research purposes. However, in clinical practice a skin biopsy may be appropriate in patients with atypical features such as systemic symptomatology, duration of lesions longer than 24 h, burning sensation rather than typical pruritic wheals that disappear over 24 h, and the presence of residual hyperpigmentation (eg, drug reactions, bullous pemphigoid, urticarial neutrophilic dermatosis (NUD), autoinflammatory syndromes, purpura, and urticarial vasculitis). CSU is a clinical diagnosis and the prediction of its prognosis, duration and response to treatment is based on many clinical and laboratory biomarkers, the application of which are still under continuous review. The typical histology for CSU shows interstitial edema with mild perivascular infiltrate of lymphocytes and relatively numerous eosinophils but sometimes there can be some neutrophils. In patients unresponsive to high dose antihistamines, a skin biopsy could be considered to exclude urticarial vasculitis and to assess the cellular infiltrates such as a mixture of eosinophils and neutrophils proposed to be a more difficult to treat form of CSU suggestive of chronic autoimmune urticaria, as this could be helpful in directing treatment approaches.^60^, ^61^, ^62^

The dominance of CD4^+^ T cell infiltration in lesional skin, especially for severe CSU patients, rather than eosinophils and basophils, might suggest that targeting T cells in these patients could be an alternative strategy, although more research is needed to support this assertion. In a recent study, increased IL-17 expression in CD4^+^ T cells in proximity with IL-17-expressing mast cells suggested the strategy of treating these patients with anti-IL-17 monoclonal antibodies which has proven to be beneficial.^54^^,^^63^ These findings suggest that IL-17 is frequently involved in the pathogenesis of some subtypes of CSU and that IL-17 should be considered a biomarker for predicting a therapeutic response.^54^^,^^63^ Future studies should explore the utility of skin biopsies in CSU patients, with the aim of assessing different histological subtypes of CSU and personalizing CSU treatments.^64^

## Immune-mediated responses, autoimmunity and related biomarkers in chronic spontaneous urticaria

For many decades, autoimmune responses were speculated to play a fundamental role in the pathogenesis of CSU, supported by its strong association of increased anti-nuclear antibodies (ANA), anti-TPO antibody and increased IgG anti-IgE antibodies.^65^ These observations resulted in the recognition of 2 autoimmune endotypes of CSU: 1) type I autoimmunity (also called autoallergy); derived by specific IgE autoantibodies against auto-antigens such as TPO, IL-24 and double strand DNA^66^ and; 2) type IIb autoimmunity characterized by the presence of IgG-specific autoantibodies, directed against IgE or α-subunit of FcεRI on mast cells and basophils, and originally assessed by the demonstration of positive ASST.^10^ In addition, other immune-mediated aspects, such as auto-reactive T-cell mediated inflammation are potentially involved. This is supported by findings of increased Th1, Th2 and Th17 cytokines in the peripheral blood of CSU patients.^67^ The prediction of CSU severity, prognosis and the response to standard treatments, mainly to H1-antihistamines and omalizumab, are assessed by using several biomarkers. The presence of ASST and anti-TPO-IgG was reported by many investigators to be associated with higher CSU severity, and a longer duration of CSU.^46^ It is important to mention that ASST is non-specific for autoimmune mediated CSU, as it can be positive in other autoimmune diseases and even in certain healthy individuals. Severe CSU (as measured by urticaria activity score (UAS7) was reported to exist in correlation with increased levels of total IgE >100 IU/ml, predicting a longer disease duration, a better response to omalizumab but is also predictive of CSU recurrence after stopping omalizumab.^68^ The lower expression of FcεRI on basophils is associated with a poor response of CSU patients to omalizumab. Circulating IgG against FcεRI in CSU patients were found to be associated with ASST positivity and the presence of anti-TPO-IgG antibodies.^69^ Currently, BAT and basophil histamine release assay, are considered reliable tests for evaluating serum autoreactivity and type IIb autoimmunity in CSU but can be performed only in laboratories with specialized equipment (Table 1) which is not available in many countries. The combination of high anti-TPO-IgG and low IgE levels was shown to exist in association with other markers of autoimmunity such as ASST and BAT, suggesting that all these markers should be included in the prediction of CSU severity, and prognosis.^70^ Of importance to note, the sensitivity and specificity of these markers have yet to be well-established as prognosticators to treatment.

If CSU is, at least in part, a T-cell mediated disease, T-cell related cytokines could be used as biomarkers for improving the diagnosis and treatment of CSU. In many studies, serum IL-33 and/or IL-17 were found to be increased in association with CSU severity.^71^ In these cases, the administration of cyclosporine A might be a better alternative in achieving a significant remission. In CSU patients in whom IL-17 expression was increased in lesional skin, anti-IL-17 therapy was shown to be significantly effective in treating patients who failed omalizumab therapy.^54^ The assessment of T-cell related biomarkers requires further evaluation.

## Conclusions

Our understanding of CSU and CIndU has significantly increased with the approval of omalizumab as phenotypes with corresponding endotypes have been more clearly defined and validated based on response or non-response to treatment. This approach has led to the association of several acute phase reactants or antibodies as biomarkers or prognosticators for treatment response. The current data support autoantibodies against IgE or FcεRI α subunit measured by basophil histamine release assays, total IgE levels and IgG autoantibodies against TPO as specific markers to differentiate autoallergic CSU from type 2b autoimmune CSU. These markers have been included as exploratory endpoints in many clinical trials investigating novel therapies or for repurposing existing biologics to determine responders and non-responders, but unfortunately, the data are still not completely clear. Therefore, further randomized controlled studies and real-world studies are needed to demonstrate more conclusively the utility of ordering these tests in CSU patients when they initially present or when it is determined they are not responsive to high dose SGAH before they can be included in evidence-based CSU guidelines. Until then, the current data support limiting diagnostic testing in patients where there is no clear history or clinical suspicion for an underlying cause which is currently suggested by current guidelines. However, serious consideration should be given to ordering IgE, basophil testing, and IgG-anti-TPO prior to starting omalizumab as these tests are less reliable once omalizumab is started. Routine ordering of biomarkers to predict optimal therapy for CSU patients may not always be feasible in different regions of the world due to economic considerations, as many countries do not have access to some of these tests which are very costly. In such cases, treatment response guided based on baseline clinical features such as patient reported outcome measures like UAS7 scores and the presence or absence of angioedema or CIndU in conjunction with ASST and available serum biomarkers will help clarify the next treatment steps after high dose SGAHs^72^ It should be noted that for more severe cases of CSU, it may be necessary to use a combination of different therapies to establish control in these patients.

## Ethics statement

No IRB approval was needed.

## Author contributions

This document was generated by the WAO Skin Allergy--Urticaria Committee. All authors contributed equally to the development and writing of this manuscript.

## Author consent to publish

All authors reviewed the final version and agreed to publication in WAO Journal.

## Availability of materials

Not applicable.

## Funding

None.

## Conflict of interest disclosure statements

JB: PI/consultant: Novartis, Genentech, Sanofi-Regeneron, Allakos, Celldex, Jasper, AZ, Amgen, Escient/Incyte, Evoimmune, Takeda/Shire, CSL Behring, Biocyrst, Kalvista, Pharvaris, Ionis, Intellia, Biomarin, Astria, Blueprint Medicine, Cogent, Telios.

IJA reports personal fees from Bayer, Bial, Cipla, Eurodrug, Gebro, Glenmark, Menarini, MSD, Roxall and Sanofi outside the submitted work.

RA has been speaker and/or consultant for Novartis, Sanofi, Jasper Therapeutcs, GSK, HAL Allergy, Allergy Therapeutics, Smart Practice.

AB: Research Grant: Intellia, Ionis, Astria; Advisory Board: CSL, Ionis, Pharvaris, Intellia, Kalvista, Astria, Takeda, ADARx.

SB: advisory Board or equivalent for Astria, Biocryst, CSL Behring, Ionis, Kalvista, Pharvaris, Sanofi, Takeda; Grants or honoraria: Biocryst, CSL Behring, Kalvista, Takeda; Participating or participated in a clinical trial: Takeda.

TC: Research-CSL Behring, Takeda, Astria, Kalvista, Pharvaris, Ionis, GSK, Sanofi; Speaking- Sanofi, Regeneron; Honorarium- CSL Behring. Takesa, Grifols, Kalvista.

RMG: Former speaker Novartis.

AB: Research Grant: Intellia, Ionis, Astria; Advisory Board: CSL, Ionis, Pharvaris, Intellia, Kalvista, Astria, Takeda, ADARx.

HJL: has participated in research with, served as a speaker or advisor for, participated in educational projects with or received educational grants from the following: Astria, CSL Behring, Intellia, KalVista, Pharvaris, Takeda.

MH: has been a speaker and/or advisor for and has received honoraria from Kaken, Kyorin, Kyowa Kirin, Mitsubishi-Tanabe, Novartis, Sanofi/Regeneron, Taiho, and Teikoku.

ASG: receives a productivity scholarship from Brazilian National Council of Research (CNPq); she received research funding, honoraria for educational activities or acted as a consultant for Catalyst Pharmaceuticals, CSL Behring, Takeda, Kalvista, Pharvaris, Pint-Pharma, Multicare, Biomarin, Binding-site, Kedrion and Astra.

DL: David Lang has carried out clinical research with, served as a consultant for, and/or has received honoraria from: AstraZeneca, Blueprint, Celldex, Genentech, Novartis, Sanofi-Regeneron.

HP: reports fees for lectures or advisory board participation (last three years) from AstraZeneca, FAES Farma, GSK, JABA Recordati, Leti, Medinfar, Menarini, Organon, Stallergenes, Tecnimede, Viatris; clinical trials paid to her Institution from Novartis and AstraZeneca.

MR: has carried out clinical research with, served as a consultant for, and/or has received honoraria from: Astria, Biocryst, Biomarin, Celldex, CSL Behring, Cycle Pharma, Grifols, Intellia, Ionis, Kalvista, Novartis, Pharming, Pharvaris, Sanofi-Regeneron, Takeda Celldex, Novartis, Sanofi-Regeneron.

SSS: NIH, Novartis, Escient, Allakos, Jasper, Celldex, Allakos, Granular Therapeutics, Genentech, Celldex, Evommune, Novartis, Escient, Celltrion, Sanofi, Nucor, GSK.

GS: has received research support from Aimmune, Amgen, Astra-Zeneca, DBV technologies, Genentech, Leo Pharma Novartis,Sanofi, Regeneron and ALK; is a medical advisor for Novartis, CSL Behring, Pfizer, Abvie, Astra-Zeneca, Nuvo Pharmaceuticals, Eli Lilly, Incyte and the Allergy Asthma and Immunology Society of Ontario.

AZ: has received honoraria, meeting/travel support, and/or served on advisory boards for Astria, BioCryst, CSL Behring, Intellia, KalVista Pharmaceuticals, Otsuka, Pharvaris, and Takeda.

TZ: reports honoraria for lectures from Amgen, AstraZeneca, AbbVie, ALK -Abelló, Almirall, Astellas, Bayer Health Care, Bencard, Berlin Chemie, FAES Farma, HAL Allergie GmbH, Henkel, Kryolan, Leti, L'Oreal, Meda, Menarini, Merck Sharp & Dohme, Novartis, Nuocor, Pfizer, Sanofi, Stallergenes, Takeda, Teva, UCB, and Uriach; Fees for industry consulting were received from Abivax, Almirall,Blueprint, Celldex, Celltrion, Novartis, and Sanofi.

BZ: Consultant - CSL Behring, Takeda/Shire, Biocyrst, Novartis, Genentech, Biomarin; Laboratory agreement – Ionis.

ML, JLGA, MMA, DVN, MIRJ, ET: nothing to report.
